# Biomechanical comparison of minimally invasive fixation for sanders II calcaneal fractures: a multi-position finite element study from rigid internal fixation to elastic kirschner wire configuration

**DOI:** 10.3389/fbioe.2026.1857728

**Published:** 2026-07-16

**Authors:** Zulong Zhou, Mingxiang Liu, Lingchao Kong, Rende Ning

**Affiliations:** Department of Orthopedics, The Third Affiliated Hospital of Anhui Medical University (The First People’s Hospital of Hefei), Anhui Medical University, Hefei, Anhui, China

**Keywords:** biomechanics, calcaneal fracture, finite element analysis, kirschner wire, minimally invasive surgery

## Abstract

**Introduction:**

Minimally invasive surgical treatment is increasingly favored for intra-articular displaced calcaneal fractures with a high risk of soft tissue complications. The calcaneus is the largest tarsal bone in the foot and plays a critical role in weight-bearing and gait mechanics; displaced fractures often result in significant functional impairment. Sanders Type II fractures, characterized by a single major fracture line that divides the posterior articular surface into two major bone fragments, are one of the most common subtypes and present a therapeutic challenge in balancing mechanical stability with soft tissue preservation.

**Methods:**

A finite element model of Sanders Type II calcaneal fractures was established to compare five fixation methods under physiological tendon loading: (1) a periosteal locking plate, (2) four hollow screws, (3) four K-wires placed along the screw trajectory, (4) two intra-articular K-wires combined with two standard K-wires, and (5) six K-wires placed at dispersed locations. Outcome measures included maximum fragment displacement, implant-induced bone stress, and implant stress.

**Results:**

Rigid internal fixation (hollow screws) provided the best initial stability and the least displacement of the bone fragments. A key finding was that under the most severe static loading condition (dorsiflexion with maximum Achilles tendon force), the maximum fragment displacement for all K-wire configurations (≤0.103 mm) was below both the clinically reported acceptable threshold (≤0.5 mm) and the safe healing range (<1 mm), indicating sufficient initial healing stability. Furthermore, K-wires generated significantly lower stress on the bone than the plate, while cannulated screw fixation carried the lowest risk of implant failure. Based on extended observations from clinical literature, K-wire fixation offers potential advantages including a minimally invasive approach, flexible handling, low implant burden, and the possibility of outpatient removal to avoid secondary surgery.

**Conclusion:**

Although rigid implants are biomechanically superior, K-wire fixation provides clinically acceptable initial stability and, combined with its minimally invasive characteristics, represents an attractive alternative. The choice of fixation method requires a comprehensive consideration of individual patient factors (such as bone quality and functional demands) and the overall treatment strategy.

## Introduction

The calcaneus is the largest tarsal bone in the foot, playing a pivotal role in weight-bearing and gait mechanics (Maceira and Monteagudo, 2015). Displaced intra-articular calcaneal fractures (DIACFs) account for 60%–75% of all calcaneal fractures, often resulting from high-energy trauma such as falls from height or motor vehicle accidents. These fractures often result in loss of calcaneal height, increased width, and irregular articular surfaces, significantly impairing patients’ walking function and quality of life (Bajammal et al., 2005; Fakhouri and Manoli, 1992). Compounding the issue, the calcaneus and perimalleolar region have poor blood supply and thin soft tissue coverage, frequently leading to severe edema, tension blisters, and even skin necrosis post-trauma. These anatomical characteristics pose significant challenges for surgical treatment: pursuing fixation strength through extensive surgical exposure may further compromise local blood supply, leading to severe complications like poor wound healing and deep infections. Therefore, the current ideal treatment approach should strive for a balance between achieving mechanical fracture stability and employing soft-tissue-friendly minimally invasive techniques, ensuring high-quality fracture reduction while minimizing postoperative risks.

Among numerous classification systems, the Sanders classification based on coronal Computed Tomography (CT) scans has become the gold standard for assessing prognosis and guiding surgical planning due to its ability to precisely describe posterior articular surface injury characteristics (Vosoughi et al., 2020). Sanders Type II fractures are particularly common clinically, characterized by the posterior articular surface being divided by a primary fracture line into two major bone fragments of comparable volume with well-defined borders. This fracture morphology dictates treatment decisions: the larger fragment provides a reliable anchor for various internal fixation techniques (plates, screws, or K-wires); however, the separation of the articular surface introduces inherent instability. Inadequate fixation may lead to postoperative secondary displacement and articular surface collapse, potentially resulting in complications like traumatic arthritis. Therefore, for Sanders II fractures, surgeons must carefully balance the robust fixation offered by traditional plates against the biocompatibility advantages of minimally invasive techniques. The core challenge lies in balancing mechanical stability with soft tissue risks. This trade-off between “mechanical reliability” and “biological preservation” constitutes the central controversy in managing this fracture type (Ma et al., 2023). Currently, primary treatment approaches for Sanders II fractures fall into two categories (Zhang et al., 2019; Sugimoto et al., 2022). One involves the traditional extended lateral approach combined with plate internal fixation, which provides reliable mechanical stability but requires extensive soft tissue dissection, carrying a higher risk of postoperative soft tissue complications and infection (Verstappen et al., 2024). The other category involves minimally invasive techniques, such as the sinus tarsi approach or percutaneous screw fixation. These methods offer the advantage of significantly reducing soft tissue trauma, thereby facilitating early patient recovery (Cao et al., 2022; Dayton et al., 2014). Therefore, clarifying the initial stability provided by different fixation methods for Sanders II fractures under minimally invasive principles holds significant clinical importance for establishing evidence-based treatment pathways and achieving personalized therapy.

Biomechanical stability is one of the key factors determining fracture healing quality and clinical efficacy (Sato et al., 2023). Although previous finite element studies have provided important references for evaluating the performance of different calcaneal fixation methods, these studies have primarily focused on rigid internal implants such as plates and screws (Ivanov et al., 2022; Lerner et al., 2005). In clinical practice, K-wire techniques are widely adopted due to their minimally invasive nature and operational flexibility. However, systematic quantitative research on their biomechanical performance, particularly stability under physiological loads, remains insufficient. Furthermore, existing models exhibit limitations in mechanical loading conditions. Most employ static axial loading, failing to adequately account for the dynamic influence of surrounding tendon forces during the gait cycle (Diniz et al., 2023). They also neglect to evaluate the potential impact of different foot positions (corresponding to distinct gait phases) on fixation outcomes. To address these research gaps, this study developed a novel finite element model integrating multiple foot positions and incorporating dynamic tendon loading. This model directly compares five representative minimally invasive fixation techniques, including transarticular K-wire fixation for Sanders II fractures. By systematically evaluating the biomechanical performance of rigid versus elastic fixation techniques and integrating this with clinical practical advantages, this analysis aims to provide a practical framework for selecting fixation strategies that balance stability and minimally invasiveness. This study focuses exclusively on biomechanical performance. No soft tissue modeling was performed, and we do not directly quantify soft tissue trauma, wound healing complications, or infection risks. Statements regarding minimally invasive advantages are based on clinical literature, not direct findings of this finite element analysis.

## Methods

### Finite element model development

**FIGURE 1:**
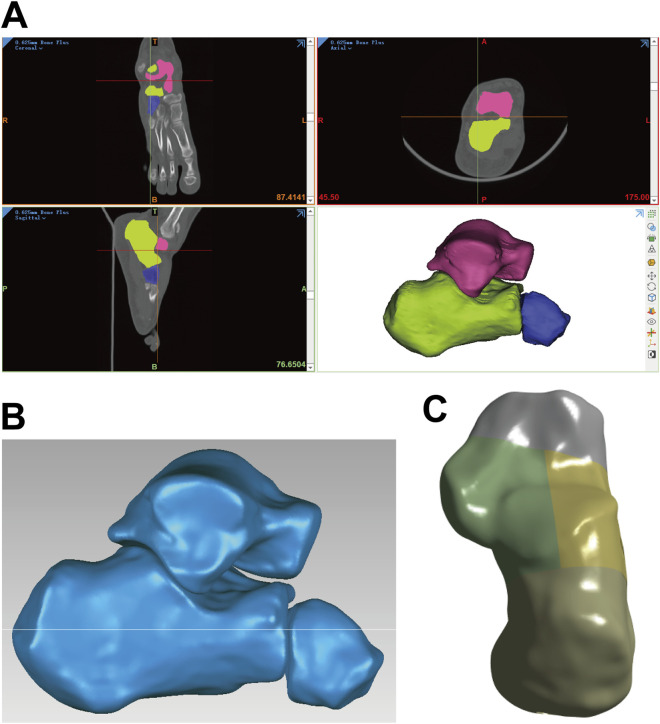
Finite element modeling process for Sanders type II calcaneal fractures: **(A)** Bone tissue segmentation and mask extraction, **(B)** Integration and reconstruction of 3D solid models, **(C)** Fracture zone partitioning and mechanical analysis preparation.

**FIGURE 2:**
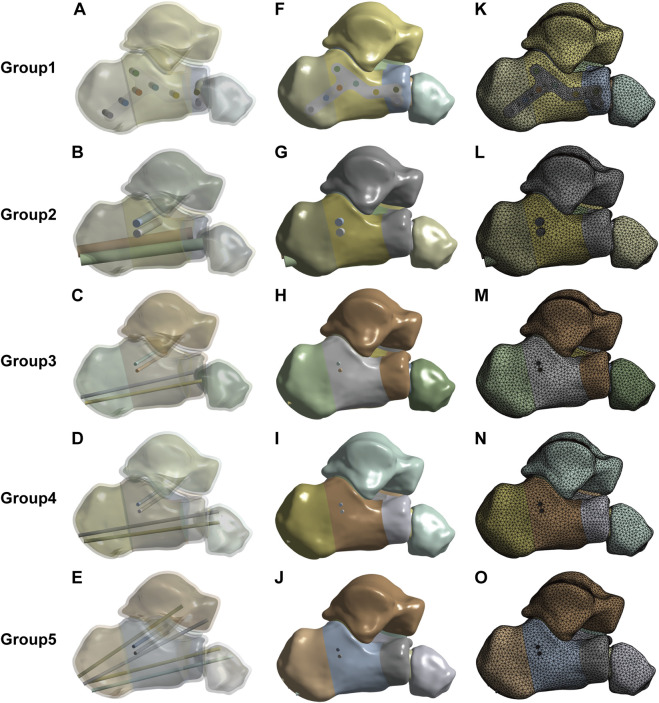
Schematic diagrams of five internal fixation techniques for Sanders type II calcaneal fractures **(A–E)** transparent views, **(F–J)** assembled models, **(K–O)** meshed models.

A high-resolution computed tomography (CT) scan (slice thickness: 0.625 mm) of a healthy adult (with no history of foot pathology) was used to reconstruct a three-dimensional (3D) model of a healthy human calcaneus. The DICOM data were imported into medical image processing software (Mimics Research 21.0, Materialise, Belgium) for segmentation and initial 3D modeling (Figure 1). Subsequently, the model underwent refinement in Geomagic Wrap 2021 (3D Systems, United States) to achieve surface smoothing and repair geometric defects. The final solid geometric model was imported into finite element analysis software (ANSYS 24.0, ANSYS Inc., United States) for assembly and meshing (Figure 2). Bone tissue was modeled as a linear elastic isotropic material with heterogeneous modulus distribution. Cortical bone was assigned an elastic modulus of 17 GPa and Poisson’s ratio of 0.3; cancellous bone was set to 0.5 GPa with a Poisson’s ratio of 0.3 (Kabel et al., 1999); articular cartilage was defined with an elastic modulus of 10 MPa and Poisson’s ratio of 0.42 (Table 1).

**TABLE 1:** Material properties assigned in the finite element model.

Material	Young’s modulus (MPa)	Poisson’s ratio
Cortical bone	17,000	0.30
Trabecular bone	500	0.30
Titanium alloy	110,000	0.30
Cartilage	10	0.42

The fracture lines were established in accordance with the Sanders IIb classification criteria (Vosoughi et al., 2020). Using the “multi-plane sectioning” function in Mimics software, we performed a virtual dissection of the calcaneal model along characteristic fracture lines based on the anatomical features of the calcaneus and the typical patterns of fracture line orientation. First, using the posterior articular surface of the calcaneus as a boundary, we created a horizontal virtual osteotomy line to separate the superior subtalar joint surface region (gray area); second, following the oblique anatomical weak zone of the Gissane angle (calcaneal groove), we drew a virtual osteotomy line running from the lateral aspect of the posterior articular surface toward the anteroinferior direction, dividing the calcaneal body into an anterolateral region (yellow area) and a posteromedial region (green area); finally, using the basal margin of the calcaneal tuberosity as a landmark, we drew a vertical virtual osteotomy line to separate the inferior calcaneal tuberosity region (light green area). The trajectory of the aforementioned virtual osteotomy lines references common injury patterns in calcaneal fractures, ensuring that the segmentation closely aligns with the distribution of actual fracture lines. A uniform 1.0 mm gap is introduced at the primary fracture line to simulate malunion of the bone ends following reduction (Figure 1).

### Fixation constructs

This study designed five representative minimally invasive fixation structures, forming a complete spectrum of fixation stiffness from rigid internal fixation to flexible K-wire techniques. The first group employed a tarsal sinus plate (Shanghai Sanyou Medical Co., Ltd.) for fixation, utilizing a low-profile locking plate conforming to the anatomical morphology of the tarsal sinus. Six 3.5 mm diameter locking screws (Sanyou Medical) were used to sequentially stabilize the posterior joint fragment, anterior protrusion, and calcaneal arch. The second group employed hollow screw fixation, utilizing four 6.5 mm partially threaded hollow screws (Sanyou Medical) implanted through the lateral calcaneal wall. These specially designed screws firmly engage calcaneal arch fragments while achieving effective interfragmentary compression. To independently evaluate the impact of fixation pathways on biomechanical performance, the third group employed pathway-mimicking K-wire fixation. This group utilized four 2.0 mm smooth K-wires, with insertion paths precisely replicating the screw trajectories of the second group. The fourth group employed transarticular K-wire fixation, configured with four 2.0 mm K-wires, two of which crossed the subtalar joint and penetrated the talus. This temporary joint crossing is a commonly used technique in clinical practice to enhance stability. The fifth group employed divergent K-wire fixation, utilizing six 2.0 mm K-wires arranged in a multiplanar divergent pattern to construct a stable three-dimensional scaffold structure (Figure 2).

All implants were simulated using a linear-elastic isotropic titanium alloy material model with a Young’s modulus of 110 GPa and a Poisson’s ratio of 0.3 (Table 1). Although titanium alloy K-wires exist clinically, it should be noted that conventional Kirschner wires used in routine practice are often made of stainless steel. The use of titanium properties for K-wires in this model may result in slightly different predicted stiffness and stress distributions compared to stainless steel wires. This material model selection aligns with the mechanical properties of the clinically prevalent Ti-6Al-4V alloy while ensuring the validity and comparability of finite element analysis. Through this experimental design, we systematically evaluated the biomechanical characteristics and differences among various fixation strategies.

### Meshing, contacts, and boundary conditions

**FIGURE 3:**
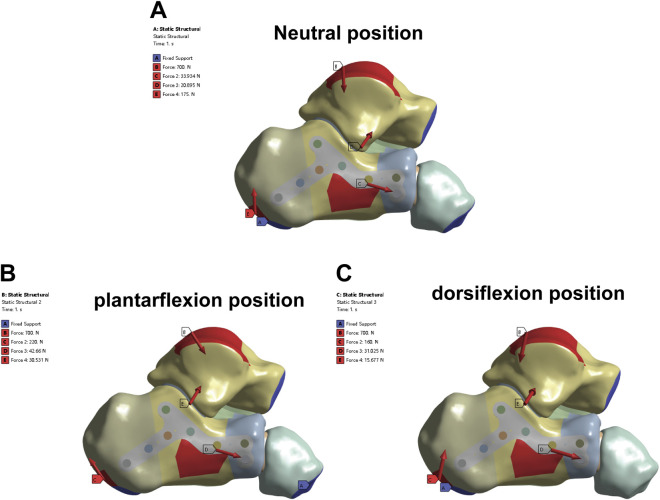
Schematic diagram of load distribution in a Sanders type II calcaneal fracture under neutral **(A)**, plantarflexion **(B)**, and dorsiflexion **(C)** positions.

The model was meshed using 10-node quadratic tetrahedral elements. Computational accuracy was ensured through mesh convergence studies. The final model’s node and element counts are detailed in Table 2, with specific ranges dependent on the implant’s structural complexity. Inter-fracture surface contact interaction was defined using a friction coefficient of 0.4 (Jörn et al., 2014). The bone-implant interface (including screws and plates) was modeled using “bonded” contact as an idealized assumption of rigid fixation, representing no relative motion between the implant and bone. This simplification is commonly used in acute fracture finite element studies and does not imply biological osseointegration, which occurs over a longer timescale. For K-wire fixation, friction contact (μ = 0.2) was defined between the wire shaft and bone surface to simulate its potential for micro-movement (Nikonovas and Harrison, 2005). For load application, concentrated forces were applied at the anatomical attachment points of the three primary tendons based on physiological data. Specific load parameters (including magnitude and direction) are configured as shown in Figure 3. Achilles tendon loading was applied using the method described in Reference (Stake et al., 2022), while peroneus longus and brevis loading was set based on research data from Reference (Ashton et al., 2024). The calcaneal articular surface was fully constrained in all degrees of freedom to accurately simulate the proximal anatomical constraint provided by the talus. This loading protocol omits body weight transmission, joint contact forces, and ligamentous constraints. The objective was to evaluate fixation stability under the most unfavorable isolated tendon loading (dorsiflexion), not to simulate the full gait cycle. This represents a conservative, worst-case assessment.

**TABLE 2:** Mesh details for the five investigated fixation constructs.

Model	Number of nodes	Number of elements
1	170,403	91,009
2	193,591	104,686
3	193,846	106,236
4	202,213	110,913
5	241,570	133,431

### Sensitivity analysis

To evaluate the impact of input parameter uncertainties on the robustness of simulation results, this study conducted a simplified single-factor sensitivity analysis on two key parameters that may influence the mechanical response of the fixation structure. The fourth fixation configuration (K-wire through the joint) under the most severe loading condition (dorsiflexion) was selected as the reference case for the sensitivity analysis.

1. Bone elastic modulus: The elastic moduli of cortical bone and cancellous bone were independently varied by ±20% from their baseline values (cortical bone: 17 GPa; cancellous bone: 0.5 GPa), and the maximum displacement of the posterior articular surface bone block was recalculated for each modulus value.
2. K-wire–bone interface friction coefficient: The friction coefficient at the interface between the smooth K-wire and bone tissue was modified from the baseline value of 0.2 to 0.1 and 0.3, respectively. The maximum displacement of the bone block under the same configuration was recalculated for each friction coefficient value.

By comparing the changes in the absolute displacement values of this configuration under different parameter settings and determining whether they remain within clinically acceptable ranges, the robustness of the study’s main conclusions to parameter variations was assessed.

### Analysis

Three independent analysis steps were established, corresponding to three simulated foot positions (neutral, plantar flexion, dorsiflexion) and their respective muscle loading conditions. All simulations employed a static general analysis workflow. These three loading scenarios were systematically simulated for five fixation configurations. Key outcome metrics included: (1) Maximum primary fragment displacement, defined as the peak displacement amplitude of the posterior articular surface fragment; (2) Peak von Mises stress within the implant, serving as an indicator of potential implant failure risk; (3) Bone stress and fracture gap displacement, representing quantitative changes in the initial 1.0 mm gap under applied physiological loading.

## Results

Tables 3–5 summarize the biomechanical characteristics of the five minimally invasive fixation constructs under three simulated foot postures. All fixation configurations exhibited the highest bone fragment displacement and implant stress under dorsiflexion; therefore, the following results are reported for this loading condition. Displacement and stress trends under neutral and plantarflexion positions were consistent with those under dorsiflexion but with lower magnitudes.

**TABLE 3:** Biomechanical properties across five models under the neutral position.

Model	Calcaneus displacement (mm)	Calcaneus stress (MPa)	Internal fixation stress (MPa)
1	0.16829	27.103	113.95
2	0.04193	13.375	8.9377
3	0.06863	10.899	68.179
4	0.03502	11.527	51.730
5	0.07063	59.996	204.06

**TABLE 4:** Biomechanical properties across five models under the plantarflexion position.

Model	Calcaneus displacement (mm)	Calcaneus stress (MPa)	Internal fixation stress (MPa)
1	0.06089	15.194	52.399
2	0.03275	14.581	5.3626
3	0.06560	13.362	45.698
4	0.01561	9.5743	29.434
5	0.05314	34.958	108.32

**TABLE 5:** Biomechanical properties across five models under the dorsiflexion position.

Model	Calcaneus displacement (mm)	Calcaneus stress (MPa)	Internal fixation stress (MPa)
1	0.22407	34.054	149.64
2	0.05144	16.327	11.329
3	0.08933	14.285	86.380
4	0.08918	14.262	87.502
5	0.10307	58.261	230.60

### Bone fragment stability

The maximum displacement of the posterior articular facet fragment was used to evaluate the stability provided by each fixation construct. Under dorsiflexion, the ranking of maximum posterior facet fragment displacement was: cannulated screws (0.051 mm) < transarticular K-wires (0.089 mm) ≈ K-wires along simulated screw trajectories (0.089 mm) < divergent K-wires (0.103 mm) < tarsal sinus plate (0.224 mm). All K-wire configurations exhibited maximum displacements ≤0.103 mm, well below the clinically reported acceptable threshold (≤0.5 mm) and the safe healing range (<1 mm) (Claes et al., 2002; Marvan et al., 2015; Li et al., 2025; Eltabbaa et al., 2023). The tarsal sinus plate group showed the largest displacement, which remained sub-millimeter (0.224 mm).

### Implant stress and failure risk

Peak von Mises stress within the implant served as a key indicator of potential failure risk. Under dorsiflexion, the ranking of peak implant von Mises stress was: cannulated screws (11.33 MPa) < transarticular K-wires (86.38 MPa) < K-wires along simulated screw trajectories (87.50 MPa) < tarsal sinus plate (149.64 MPa) < divergent K-wires (230.60 MPa). The cannulated screw group exhibited extremely low stress, indicating the lowest risk of implant failure. The divergent K-wire group showed the highest stress (230.60 MPa), suggesting a potential risk of bending or breakage in clinical application. The transarticular K-wire and simulated screw trajectory K-wire groups demonstrated moderate stress levels.

### Bone stress and fracture gap changes

Stress distribution within the calcaneus and fracture gap motion provided additional insights. Under dorsiflexion, the ranking of peak bone stress was: transarticular K-wires (14.26 MPa) < K-wires along simulated screw trajectories (14.29 MPa) < cannulated screws (16.33 MPa) < tarsal sinus plate (34.05 MPa) < divergent K-wires (58.26 MPa). Divergent K-wires and the tarsal sinus plate led to higher bone stress concentration, whereas the other three groups exhibited lower and more uniform bone stress distribution. Regarding fracture gap displacement, all constructs successfully limited post-loading changes of the initial 1.0 mm gap to within 0.05 mm, with no clinically significant differences among groups.

### Sensitivity analysis results

The results of the univariate sensitivity analysis for bone elastic modulus and the friction coefficient at the K-wire–bone interface are as follows.

1. Effect of bone elastic modulus: When the elastic modulus of cortical bone and cancellous bone increased by 20%, the maximum displacement of the transarticular K-wire group in the dorsiflexion position was 0.0721 mm; when the elastic modulus decreased by 20%, the maximum displacement was 0.1140 mm (baseline value: 0.089 mm). Within the aforementioned range of parameter variations, displacement values ranged from 0.07 to 0.12 mm, all of which were well below the clinically safe healing threshold reported in the literature (<1 mm).
2. Effect of the K-wire–bone interface friction coefficient: When the friction coefficient was set to 0.1, the maximum displacement was 0.0990 mm; when set to 0.3, the maximum displacement was 0.0919 mm (baseline value: 0.089 mm). Displacements under different friction coefficient values were all close to the baseline value and remained well below the clinically safe healing threshold.

Overall, this sensitivity analysis indicates that while reasonable fluctuations in input parameters cause the absolute displacement to vary within a certain range, the maximum displacement under all parameter combinations remains well below the clinically acceptable safety threshold for healing (<1 mm). Therefore, the core conclusion of this study—that “K-wire fixation provides clinically acceptable initial stability”—exhibits relative robustness to reasonable parameter variations.

## Discussion

Regarding minimally invasive fixation strategies for Sanders type II calcaneal fractures, the characteristics and indications of different implants are central to the discussion. This study selected the Sanders IIb subtype as the research object because its fracture line runs sagittally along the posterior articular facet, forming two mediolateral bone segments of similar volume, which presents typical biomechanical characteristics among type II fractures. Compared with type IIa (fracture line lateral) and type IIc (fracture line medial), the centrally located fracture line of type IIb better reflects the stability challenge of sagittal splitting of the posterior facet. It should be clarified that this study only evaluated the initial stability of each fixation method under acute loading conditions and did not simulate cyclic loading or assess fatigue performance.

This study quantitatively compared the initial biomechanical performance of five minimally invasive fixation techniques under physiological tendon loading using finite element analysis. The data showed that cannulated screws provided the best overall stiffness and implant reliability (displacement of 0.051 mm, stress of 11.33 MPa under dorsiflexion). A key finding was that all K-wire configurations exhibited maximum posterior facet fragment displacements ≤0.103 mm under dorsiflexion, well below the clinically acceptable threshold (≤0.5 mm) and safe healing range (<1 mm) cited in this study (Augat et al., 2021; Buckley et al., 2002; Claes et al., 2002). This finding revises the traditional understanding of K-wire fixation efficacy, demonstrating that K-wires can provide clinically acceptable initial stability under specific fracture morphologies and mechanical conditions, rather than being “mechanically equivalent” to rigid fixation. Therefore, when developing a fixation strategy, a comprehensive framework integrating biomechanical performance with clinical factors (such as minimally invasive approach, soft tissue protection, surgical flexibility) should be established (Sugimoto et al., 2022; Dayton et al., 2014). Within this framework, K-wires demonstrate unique clinical value: they overcome the geometric limitations of conventional implants. Unlike locking plates that offer only pre-set fixed angles, K-wires allow surgeons to achieve individualized multiplanar, multi-angle placement according to fracture line direction and fragment morphology. More critically, they enable transarticular fixation—something difficult to safely achieve with cannulated screws. When cannulated screws are used for transarticular fixation, their continuous rigid compression may lead to mechanical wear of articular cartilage and disruption of subchondral blood supply, whereas the elastic fixation characteristics of K-wires provide moderate stability while allowing early functional micromotion at the joint. This unique advantage has important clinical implications when managing complex fractures involving the subtalar joint.

**FIGURE 4:**
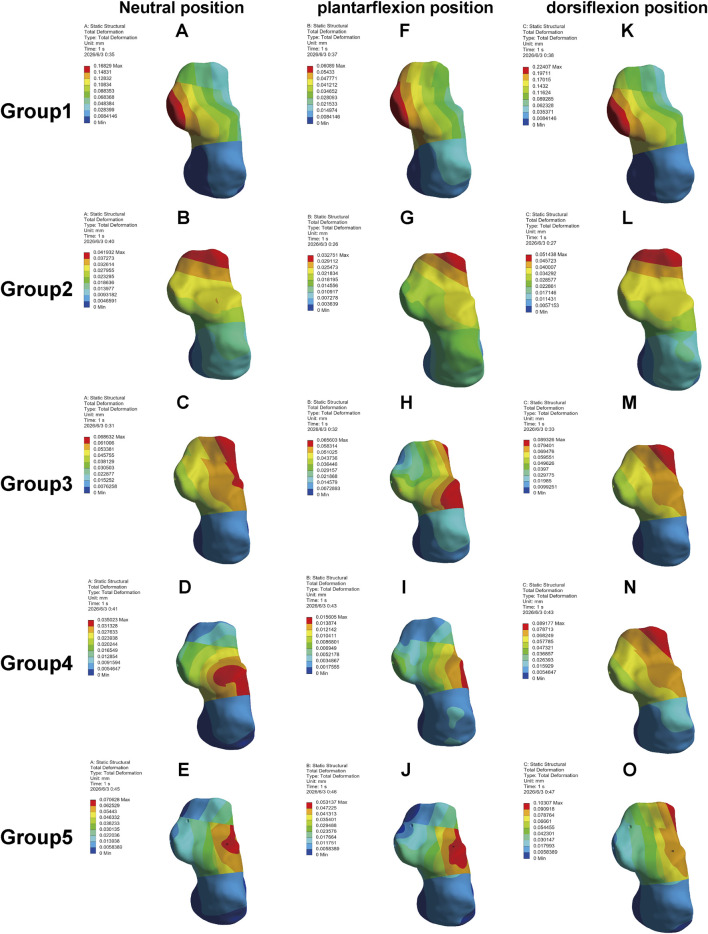
Displacement contours demonstrating five internal fixation techniques for Sanders type II calcaneal fractures under neutral **(A–E)**, plantarflexion **(F–J)**, and dorsiflexion **(K–O)** loading.

**FIGURE 5:**
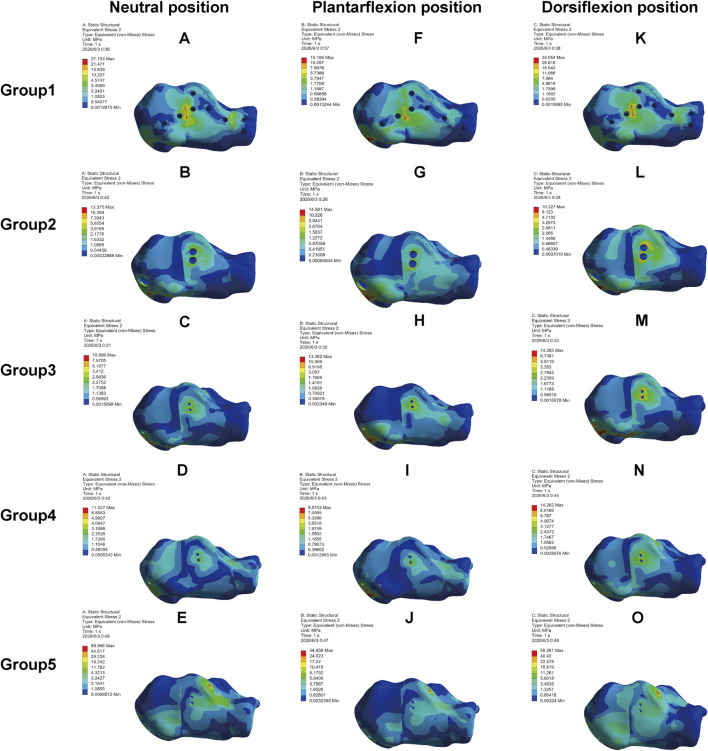
Stress distribution for five internal fixations of a Sanders type II calcaneal fracture under neutral **(A–E)**, plantarflexion **(F–J)**, and dorsiflexion **(K–O)** positions.

**FIGURE 6:**
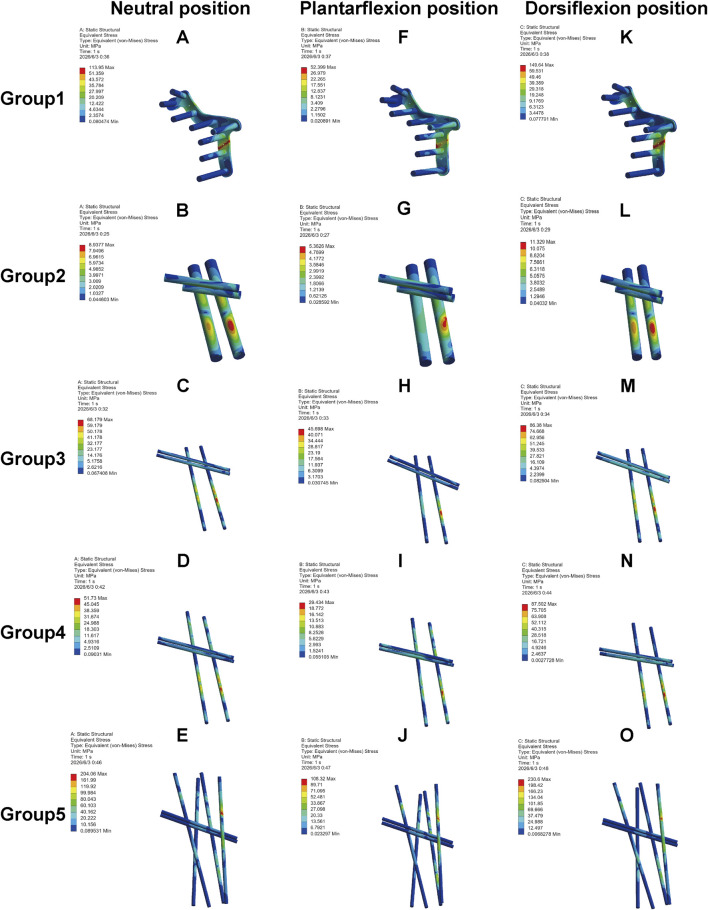
Implant stress contours for five internal fixations of a Sanders type II calcaneal fracture under neutral **(A–E)**, plantarflexion **(F–J)**, and dorsiflexion **(K–O)** positions.

Performance differences among implants of varying stiffness provide important insights. The second group exhibited excellent stability under dorsiflexion, with a maximum posterior facet fragment displacement of only 0.051 mm and a peak implant stress as low as 11.33 MPa (Figures 4, 5). This confirms that interfragmentary compression fixation is the gold standard for achieving absolute stability (Ivanov et al., 2022; Jiang et al., 2024). This superior performance stems from its low-profile design and efficient load distribution/transfer mechanism. In contrast, the first group (tarsal sinus plate) exhibited the largest fragment displacement (0.224 mm) and relatively high peak implant stress (149.64 MPa) under dorsiflexion. This reveals the double-edged sword effect of overemphasizing rigidity: its highly rigid structure may induce “stress shielding” at the bone–implant interface (Figure 6), where elastic modulus mismatches lead to greater deformation under system loading (Li et al., 2022). This phenomenon reminds clinicians to exercise caution when selecting fixation for periarticular fractures. For certain fracture types, moderately stiff K-wire fixation—combined with its well-documented minimally invasive characteristics in clinical practice—may offer a more optimized solution than excessively rigid fixation.

The core contribution of this study lies in providing extensive biomechanical data supporting the precise and rational application of K-wire techniques. The data show that K-wires can limit fragment displacement to the sub-millimeter level (Figure 4): under dorsiflexion, displacements for groups 3, 4, and five were 0.089 mm, 0.089 mm, and 0.103 mm, respectively. Theoretically, based on prior mechanobiological literature, this micromotion level may promote callus formation through mechanobiological signaling, although this effect is dose-dependent, with excessive micromotion (typically >1 mm) potentially leading to fibrous union or nonunion (Augat et al., 2021; Claes and Cunningham, 2009; Yamaji et al., 2001; Epari et al., 2006). It should be clarified that our model did not directly simulate or measure any biological responses. The displacement range observed in this study (0.051–0.224 mm) lies at the lower end of this spectrum, but it must be emphasized that one cannot directly infer from this model that such micromotions necessarily produce a biological promotional effect. The core mechanism may involve: appropriate cyclic micromotion induces controlled mechanical strain in the periosteal region, which is sensed by local mechanoreceptors, initiating mechanotransduction pathways that convert mechanical signals into cascading biochemical osteogenic signals, ultimately forming new bone through orderly mineralization. This biological response mechanism, consistent with Wolff’s law, provides theoretical support for the advantages of elastic fixation techniques in promoting fracture healing. More importantly, compared with the plate group (bone stress of 34.05 MPa under dorsiflexion) and the divergent K-wire group (58.26 MPa under the same condition), certain K-wire groups (groups 3 and 4) produced lower and more uniformly distributed bone stress within the calcaneus, with stress values consistently below 15 MPa under dorsiflexion. This “low-stress, low-interference” characteristic, combined with the excellent soft tissue protection known from percutaneous insertion techniques in clinical practice, may theoretically contribute to a more favorable biological environment for fracture healing (Augat et al., 2021; Claes et al., 2002). This is particularly critical in the calcaneal region, which has fragile blood supply and a high risk of wound complications (Verstappen et al., 2024).

Performance differences among K-wire configurations demonstrate the flexibility and strategic applicability of this technique. The fourth group (transarticular K-wires) achieved stability comparable to screws (displacement 0.089 mm) with moderate implant stress values (87.50 MPa) (Figure 5). This strongly supports its use as an effective temporary transitional fixation or as an enhancement to initial fixation in complex fractures (Ferreira et al., 2024). The fifth group (divergent K-wires) produced the highest implant stress under dorsiflexion (230.60 MPa), suggesting a potential risk of bending or breakage in clinical use. However, its multiplanar “scaffold” effect demonstrates unique value when managing highly comminuted fragments that are difficult to stabilize individually. The third group (K-wires along simulated screw trajectories) reveals a key principle: when implant configurations are similar, the difference in displacement (K-wires 0.089 mm vs. screws 0.051 mm) is smaller than the difference in stiffness, indicating that the implant placement strategy itself is an important source of stability, not solely dependent on material stiffness. From a clinical perspective, K-wires also offer additional advantages such as reduced material costs and ease of outpatient removal, but these are extended observations based on clinical literature and are not direct conclusions of this finite element analysis (Marvan et al., 2015; Li et al., 2025; Eltabbaa et al., 2023). It must be objectively recognized that K-wires have mechanical limitations: their high stress risk necessitates appropriate postoperative protection and timely removal planning.

The data from this study preliminarily support the adoption of individualized, stratified fixation strategies in clinical practice (Rammelt and Swords, 2021). For patients with good bone quality, favorable soft tissue conditions, and a need for early aggressive rehabilitation, cannulated screws are undoubtedly the first choice due to their superior initial stability and reliability (Raza et al., 2024). Calcaneal plates offer angular stability, but their associated potential risks—such as potentially inducing greater displacement and soft tissue irritation—must be carefully weighed in decision-making (Goldzak et al., 2014). K-wires should be regarded as a highly valuable fixation method for specific indications (Marvan et al., 2015). Based on clinical literature, they demonstrate unique value in the staged treatment of open fractures: as temporary fixation after initial debridement, their minimally invasive characteristics maximize protection of damaged soft tissues and blood supply. If subsequent clinical evaluation confirms fracture stability and absence of deep infection, this temporary fixation can serve as definitive treatment. After fracture healing, K-wires can be removed directly in an outpatient setting, completely avoiding a return to the operating room for a second implant removal surgery. Furthermore, the value of K-wires in cost-effectiveness and healthcare resource optimization should not be overlooked—they significantly reduce material costs, alleviating financial burdens on patients and economically constrained healthcare systems. Surgeon decision-making should be based on a comprehensive three-dimensional assessment of fracture morphology, patient factors, and healthcare resources, rather than rigidly adhering to claims of absolute superiority of any single technique. Beyond comparing fixation constructs themselves, our multi-position loading simulation provides deeper insights into understanding the biomechanical behavior of the calcaneus under dynamic muscle control. Across all groups, dorsiflexion exhibited the highest fragment displacement and implant stress, indicating that this position simulates the most mechanically demanding phase of the gait cycle. This phenomenon is primarily driven by maximal Achilles tendon tension, generating a strong torque that pulls the calcaneal tuberosity fragment, thereby challenging the fixation system’s ability to maintain articular surface reduction. In contrast, neutral and plantarflexion positions presented relatively lower mechanical loading environments. This load-dependent performance gradient provides important considerations for postoperative rehabilitation: consciously avoiding forceful dorsiflexion movements in early rehabilitation protocols can protect the fixation construct during the initial healing phase. This strategy is particularly important when applying elastic fixation methods such as K-wires, because although these techniques provide clinically acceptable stability, such strategic load management can further optimize the healing environment (Postolka et al., 2024).

It must be emphasized that this study is a finite element analysis and does not assess actual biological healing, vascular changes, or clinical outcomes. The above interpretations regarding biological effects are entirely theoretical extrapolations based on published mechanobiological principles and are not direct findings of this study. This study has inherent limitations associated with finite element analysis. The linear elastic isotropic assumption of bone tissue cannot fully simulate the complex mechanical behavior of real bone; acute static loading cannot predict implant fatigue life under cyclic loading; complete constraint of the calcaneal articular surface neglects subtalar joint motion, potentially overestimating overall structural stability; all implants were modeled as titanium alloy, whereas clinical K-wires are frequently made of stainless steel, this may influence absolute stress values, although the comparative ranking between fixation methods is unlikely to change; simplified muscle loading and a single fracture model also limit the external validity of the results. Additionally, body weight transmission, joint contact forces, and ligamentous constraints were not modeled. Their absence may overestimate fragment displacement, as joint compression would otherwise provide stability. Results should therefore be interpreted as a conservative benchmark. Furthermore, this study did not incorporate any soft tissue models and therefore could not quantify the soft tissue trauma, wound healing complications, or infection risks associated with each fixation method. The use of a healthy bone model also limits extrapolation of results to compromised bone conditions such as osteoporosis. Future research should incorporate a complete ankle-subtalar joint model including the talus, tibia, fibula, major ligaments, and physiological joint contact forces, and use dynamic gait simulation with time-varying muscle forces and ground reaction forces. Additionally, more realistic bone tissue models, probabilistic methods to assess the impact of anatomical and biological variability on outcomes, and parametric analyses targeting different bone densities would further improve clinical relevance. This will generate biomechanical insights that more closely approximate clinical reality.

## Conclusion

In summary, this finite element analysis demonstrates that, under acute loading conditions, hollow screw fixation provides the most superior initial biomechanical performance (minimal bone fragment displacement and lowest implant stress) for Sanders Type II calcaneal fractures. All evaluated K-wire fixation techniques provided clinically acceptable initial stability (maximum bone fragment displacement ≤0.103 mm, well below the clinically acceptable threshold of ≤0.5 mm), while offering potential advantages such as minimally invasive procedures, soft tissue protection, and flexible needle placement. Furthermore, based on clinical practice, K-wires offer advantages such as outpatient removal and the avoidance of secondary surgery. Therefore, when selecting the optimal fixation strategy in clinical decision-making, one should not simply pursue maximum theoretical rigidity, but rather carefully balance biomechanical evidence, the patient’s individualized clinical condition (such as bone quality and functional requirements), and overall healthcare resource efficiency.

## Data Availability

The raw data supporting the conclusions of this article will be made available by the authors, without undue reservation.
